# Effects of a Low-FODMAP Diet on Irritable Bowel Syndrome in Both Children and Adults—A Narrative Review

**DOI:** 10.3390/nu15102295

**Published:** 2023-05-13

**Authors:** Ionela-Daniela Morariu, Liliana Avasilcai, Madalina Vieriu, Vasile Valeriu Lupu, Branco-Adrian Morariu, Ancuța Lupu, Paula-Cristina Morariu, Oana-Lelia Pop, Iuliana Magalena Starcea, Laura Trandafir

**Affiliations:** 1 Department of Environmental and Food Chemistry, “Grigore T. Popa” University of Medicine and Pharmacy, 700115 Iasi, Romania; ionela.morariu@umfiasi.ro (I.-D.M.);; 2 Department of Analytical Chemistry, “Grigore T. Popa” University of Medicine and Pharmacy, 700115 Iasi, Romania; 3 Department of Mother and Child, “Grigore T. Popa” University of Medicine and Pharmacy, 700115 Iasi, Romania; 4 Department of Pharmacology, “Sfântul Spiridon” Clinical Emergency Hospital, 700111 Iasi, Romania; 5 Department of Internal Medicine, “Sfântul Spiridon” Clinical Emergency Hospital, 700111 Iasi, Romania; 6 Department of Food Science, University of Agricultural Sciences and Veterinary Medicine, 400372 Cluj-Napoca, Romania; 7 Molecular Nutrition and Proteomics Lab, CDS3, Life Sciences Institute, University of Agricultural Sciences and Veterinary Medicine, 400372 Cluj-Napoca, Romania; 8 Pediatric Nephrology Department, “Grigore T. Popa” University of Medicine and Pharmacy, 700115 Iasi, Romania

**Keywords:** irritable bowel syndrome, low-FODMAP diet, oligosaccharides, disaccharides, monosaccharides

## Abstract

Irritable bowel syndrome is a typical gastrointestinal disease that causes bloating, flatulence, abdominal pain, diarrhoea, constipation, or alteration of the last two in adults and children. A diet low in fermentable oligosaccharides, disaccharides, monosaccharides, and polyols (FODMAP) is one of the potential treatment strategies to reduce abdominal symptoms and increase the quality of life. The present narrative review aims to present a general overview of current studies that have evaluated the efficacy of a low-FODMAP diet against other diets in gastrointestinal symptoms, nutrient intake in adults and children, and lifestyle quality. The research was performed using seven searchable databases, which included the Cochrane Central Register of Controlled Trials (CENTRAL), Cochrane Database of Systematic Reviews (CDSR), Excerpta Medica Database (EMBASE), Medline, PubMed, Scopus, and Web of Science, up to March 2023. In conclusion, there is significant evidence that the follow-up of a low-FODMAP diet might be a feasible first-line therapeutic strategy to reduce stomach discomfort, pain, bloating, and quality of life for patients with irritable bowel syndrome.

## 1. Introduction

Irritable bowel syndrome (IBS) is a frequent functional disorder of the gastrointestinal tract (GI) designated by the Rome IV diagnostic criteria, considering the increase in the number of daily cases, as there are currently more than 3.9 million female patients and more than 3.0 million male patients who present this pathology worldwide [1]. IBS causes changes in bowel habits in terms of diarrhoea and/or constipation, abdominal pain, bloating, and flatulence in adults and children [2,3]. At the same time, it causes a decrease in quality of life (QoL) [4], labour productivity, and higher care costs [5].

The increased incidence of diseases, especially among women, demands a thorough study of the production mechanisms [6]. Even if the pathophysiological mechanism remains incompletely revealed, altered GI motility, visceral hypersensitivity, intestinal microbiota imbalance [7], altered brain–gut axis [8], inflammation of the digestive tract, and psychological factors appear to determine the occurrence and development of IBS [9].

A new treatment option for IBS is the low-FODMAP diet (LFD). Following that diet reduced the specific symptoms of IBS [10,11,12,13]. Excluding certain components of the diet could be a viable option for the nutritional management of IBS. Food products that aggravate the symptoms in most patients with IBS are those that contain lactose (dairy products), fructose (oranges, dates, cherries, apples, and pears), and sweeteners (sorbitol, mannitol, and xylitol) [14,15].

The novelty of this paper is stated in the comprehensive review of current studies that have evaluated the efficacy of a low-FODMAP diet against other diets in both adults and children with irritable bowel syndrome (IBS).

Our study aims to provide a general overview of the effects of a low-FODMAP diet on gastrointestinal symptoms, nutrient intake, and lifestyle quality, making it a useful resource for clinicians and researchers working in this field. Furthermore, the study suggests that a low-FODMAP diet may be a feasible first-line therapeutic strategy to reduce stomach discomfort, pain, bloating, and improve quality of life for patients with IBS.

## 2. Materials and Methods

The research team conducted a thorough investigation of the effectiveness of the LFD in IBS treatment using 7 searchable databases: Cochrane Central Register of Controlled Trials (CENTRAL), Cochrane Database of Systematic Reviews (CDSR), Excerpta Medica Database (EMBASE), Medline, PubMed, Scopus, and Web of Science up to March 2023.

The search terms used were: “irritable bowel syndrome”, “irritable colon”, “fructose oligosaccharide”, “FODMAP or FODMAPs”, “diet restriction”, “carbohydrate diet”, “clinical trials”, “double-blind”, “blind”, “randomised controlled trials”, “meta-analysis”, etc. The search process was not limited to English.

The following criteria were stated as the study protocol: (1) randomised controlled trials (including cross-over trials), (2) patients older than 4 years, (3) Rome I, II, III, or IV diagnostic criteria, (4) effectiveness of LFD, (5) comparing LFD with a placebo/regular diet, (6) results such as reduction in IBS symptoms, improvement in QoL, and stool regularity/frequency. Exclusion criteria for the trials selected were those that included patients with IBD, dementia, diabetes, renal, cardiovascular, and hepatic disease, patients with previous GI surgery, patients using antibiotics, prebiotics, probiotics or narcotics, and patients with food allergies.

## 3. Irritable Bowel Syndrome

### 3.1. Overview

IBS is a widespread functional GI disorder that determines symptoms such as chronic abdominal pain, flatulence, bloating, and altered bowel habits [16,17]. Depending on diagnostic standards and the regional area, this pathology has a prevalence between 5% and 20% in adults [18,19,20]. IBS can occur among patients of any age, even among children, more precisely 13.5% worldwide [21], and adolescents, rarely manifesting in older patients. IBS has a slightly higher prevalence among women than males and between 18 and 39 years of age [18,19].

Although there is currently no specific biomarker for IBS, the diagnosis was established based on clinical history. Until 2006, diagnosing it seemed difficult for doctors because symptoms could change over time, but with the formulation of diagnostic criteria, the work of physicians became easier. Based on Rome IV diagnostic criteria and their most recent revision in 2016, IBS represents recurrent abdominal pain, which occurred weekly three months prior, coupled with a minimum of two of the subsequent criteria: influenced by bowel movements, associated with changes in the frequency and/or appearance. Following that classification, patients are grouped into three categories according to the pattern of the most frequent bowel movements: IBS with constipation (IBS-C), IBS with diarrhoea (IBS-D), IBS with mixed bowel habits (IBS-M), or IBS unclassified (IBS-U) [22].

### 3.2. Pathophysiology

The pathophysiology of IBS is very complex and still incompletely understood, as it involves altered enteric neurotransmitters, intestinal microbiota imbalances, neuroendocrine disorders, visceral hypersensitivity, changes in intestinal barrier function, and changes in motility and the response to maladaptive stress response [23,24]. It has been found to be an alteration of bidirectional communication through the brain–intestinal axis caused by an intricate association of biological, psychological, and social variables that underlie the condition. Communication between the brain and the gut is mediated by the autonomic nervous system. A decrease in parasympathetic activity and an increase in sympathetic nervous system activity are frequently observed in patients with IBS. The decrease in vagal tone, which influences peripheral inflammation and permeability, as well as gastrointestinal motility and sensitivity, can be caused by stress [25]. On the contrary, the vagus nerve can indirectly detect the gut microenvironment and transmit this information to the brain [26,27].

Bacterial overgrowth in the small intestine in the majority of patients provides evidence that gut microbiota is at the forefront of the pathophysiology of IBS [23]. Bloating, constipation, diarrhoea, and flatulence are the main symptoms of intestinal bacterial overgrowth. In approximately 25% of patients, the onset of IBS precedes an enteric infection [28]. As a result, post-infectious IBS is a subtype of enteric pathology dominated by diarrhoea, with a high risk of acquisition in women with severe enteritis or after prolonged antibiotic treatment. The intestinal tract presents an increased number of T cells in the lamina propria, intraepithelial lymphocytes, mast cells at the mucosa level and enteroendocrine cells containing serotonin, thus sustaining the development of a pro-inflammatory environment. In chronic inflammation, juxtaposing mast cell mediators with enteric nerves contributes to the visceral hypersensitivity seen in post-infectious IBS [29].

Food is an additional element that contributes to the pathophysiology of IBS [30,31]. Short-chain carbohydrate fermentation reveals the process through which enteric bacteria and the presence of food allergies, nonimmune food sensitivities, changes in gut hormones, and changes in the gut microbiome produce symptoms of IBS. The use of non-steroidal anti-inflammatory drugs (NSAIDs), antibiotics, infections, and stress are known triggers for IBS symptoms. However, [11] ingesting foods high in FODMAPs and foods high in biogenic amines, which produce histamine [10,30], has been associated with the onset of gastrointestinal symptoms in IBS [11,12,29].

However, it has been found that early life experiences (such as dysfunctional family factors and trauma from psychological and physical abuse) are linked to IBS susceptibility. Anxiety and depression influence pain sensitivity, gut motility, immune function, and QoL [27,29,32,33].

### 3.3. Diagnosis

The diagnosis of IBS requires the presence of characteristic symptoms within the last 3 months and the appearance 6 months ago. The Bristol stool form scale can help with the problematic subtyping of IBS because it is based on stool form [2,34,35].

The diagnosis of IBS is made after complete anamnesis based on the characteristic symptoms and results of various preliminary laboratory analyses, including complete blood count (CBC), determination of C-reactive protein (CRP), rapid erythrocyte sedimentation rate (ESR), and serological tests for coeliac disease [26,36,37,38].

Faecal lactoferrin (FL) and faecal calprotectin (fCal) are two biomarkers of intestinal inflammation that are useful for diagnosis. Their analysis is superior to serological tests (e.g., ESR and CRP) for differentiating inflammatory bowel disease (IBD) from IBS. Studies showed that measuring fCal in IBS led to a 67% reduction in the number of adults that require a colonoscopy. The determination of fCal in patients less than 45 years old is necessary to rule out IBD.

Although not widely available, rapid testing is available for both FL and fCal. The combination of CRP and fCal tests provides an even greater discrimination of IBS from IBD [39,40]. 

However, fCal is not a definitive marker for the diagnosis of IBD and may be elevated in obesity, infection, malignancy, or due to certain drugs (e.g., proton pump inhibitors or non-steroidal anti-inflammatory drugs) [36].

The lysozyme, polymorphonuclear neutrophil elastase, neutrophil lipocalin, and myeloperoxidase are other faecal proteins that have been investigated as biomarkers in IBS. Because these were limited studies, their relevance to the diagnosis of IBS is still uncertain [39].

To rule out other symptoms, a digital abdominal and rectal examination is required. This could confirm stool consistency, including rectal impaction, and it can detect dyssynergic defecation (paradoxical contraction on rectal examination during exertion) or low rectal masses [36].

Endoscopy is the ‘golden investigation’ for diagnosis of diseases of the gastrointestinal tract. It allows direct visualisation and offers the possibility of performing biopsies and establishing a histological diagnosis. However, despite those benefits, it is unpleasant to patients and may cause complications. Therefore, simple, non-invasive, and cheap tests to distinguish between intestinal diseases are beneficial [39].

For IBS, it is important to perform colon cancer screening with the help of colonoscopy. Colonoscopy is a frequent test performed to determine whether a disease, such as IBD, microscopic colitis, or colon cancer, is not the cause of a patient’s digestive symptoms. Polyps, haemorrhoids, and diverticula are just some of the lesions identified in patients with IBS during colonoscopy [26,38].

### 3.4. Therapeutic and Nutritional Management

**Figure 1:**
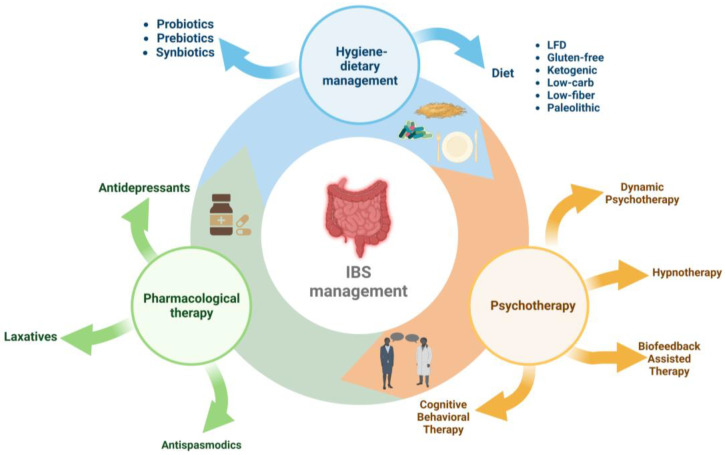
Therapeutic and nutritional management of irritable bowel syndrome.

Management of IBS includes three directions (Figure 1): pharmacological therapy (antidepressants, antispasmodics, and laxatives), interventions on hygienic-dietary revitalisation [41,42,43], and psychotherapy (cognitive behavioural psychotherapy, dynamic psychotherapy, hypnotherapy, and biofeedback-assisted stress management intervention) [10,26,44].

During the previous ten years, there has been an increase in interest in changing the lifestyle and the hygienic dietary regimen, with patients opting for one of the following diets: LFD, gluten-free diet, low-fibre diet, low-carb diet, ketogenic diet, and palaeolithic diet [22,45,46].

The LFD is a hygienic-dietary intervention option that promotes the intake of foods that include reduced amounts of fermentable oligosaccharides, monosaccharides, and polyols. It improves global symptoms, abdominal pain, bloating, bowel water content, and QoL [47,48,49,50].

## 4. Low-FODMAP Diet

The LFD is one of the most common nonpharmacological treatments for IBS. The acronym FODMAP stands for all foods that contain fermentable oligosaccharides, disaccharides, monosaccharides, and polyphenols [51]. These include fruits, vegetables, dairy, and cereals that contain short-chain carbohydrates that are harder to digest [47,52]. They can produce gas through intestinal microbial fermentation, particularly in the colon, and increased water retention via osmosis in the small intestine and colon due to insufficient absorption in the small intestine. Short-chain carbohydrates ferment quickly, producing hydrogen, carbon dioxide, and methane. The other important fermentation products are short-chain fatty acids, which enhance motility by enabling sodium and water absorption. Thus, luminal distention occurs through increased gas output and luminal water retention [53]. In susceptible individuals, these mechanisms produce luminal distension and characteristic GI symptoms, particularly gas production [53].

High intake of FODMAPs is also linked to visceral hypersensitivity, inflammation, intestinal barrier dysfunction, dysbiosis, and other conditions related to the pathogenesis and worsening of IBS [29].

Any food that exceeds any of the following amounts is considered high in FODMAPs: more than 4 g of lactose; more than 0.3 g of 0.3 g of mannitol; more than 0.3 g sorbitol; more than 0.3 g of galacto-oligosaccharides; more than 0.3 g of fructans if grain-based, otherwise, more than 0.2 g of fructans for grain-free products or more than 0.2 g of fructose [29].

Clinical research has shown the usefulness of the LFD, revealing that a restriction of FODMAP improved IBS symptoms in 70% of subjects [54]. In addition to the advantages obtained from following that diet, some disadvantages have also been reported: the complexity of diet monitoring among patients, the limitation of a certain food, the high costs, and the need for monitoring by a nutritionist to ensure an optimal nutrition intake.

Depending on the FODMAP content, the products are split into two categories: high-FODMAP foods versus low-FODMAP foods, as shown in Table 1.

FODMAP occurs naturally in various foods that contain oligosaccharides and disaccharides (e.g., dairy products), such as fructans (e.g., garlic and onion), galacto-oligosaccharides (e.g., vegetables), and monosaccharides (e.g., honey), but also polyols used as sweeteners (e.g., sorbitol, mannitol, and xylitol). The amount of FODMAP is dependent on the species and the maturity of the product [55,56,57,58].

Implementing the LFD requires the guidance of specialists (nutritionists and gastroenterologists) because it requires careful guidance during each of its three phases. The first is the elimination phase, which involves eliminating FODMAP-rich products from the diet for 3–6 weeks. The results are already seen after 1–2 weeks from the start of the diet. The second phase is represented by the gradual reintroduction of foods containing high amounts of FODMAP. In the last stage, the diet is customised for each patient for the long term [48,54,59].

## 5. Results and Discussion

### 5.1. Results and Discussion in Adults

In the scientific literature, we have identified 15 randomised control trials (RCTs) in adults using the Cochrane Central Register of Controlled Trials (CENTRAL), the Cochrane Database of Systematic Reviews (CDSR), the Excerpta Medica Database (EMBASE), Medline, PubMed, Scopus, and Web of Science. In Table 2, we summarise all the characteristics of those studies.

#### 5.1.1. Effects on Global Symptoms, Abdominal Pain, and Bloating in Adults

The LFD had favourable effects on IBS symptoms, particularly in relieving abdominal pain, bloating, and diarrhoea [49]. Furthermore, there was an improvement in intestinal movements and stool characteristics for those who followed a LFD [13,75].

After LFD intervention, Wong et al. [73], reported that 68% of patients with IBS had improved GI symptoms, noticeable even after the first week of the diet. Among the most common symptoms of IBS, abdominal pain decreased by 60%, bloating by 70%, and flatulence by 87.5%. Regarding stool formation, those with IBS-D had a more significant improvement.

The analysis performed by Bohn et al. [62] highlighted an improvement in global symptoms among patients who followed the LFD compared with the traditional IBS diet. Since the 29th day, a significant improvement compared with the baseline value for the frequency and intensity of abdominal pain was observed in the group who followed the LFD. Unlike the baseline value, there was a statistically significant reduction in the number of bowel movements in the LFD group (*p* < 0.0001), whereas there was none in the traditional IBS diet group. Furthermore, according to Zahedi et al. [74], compared with general dietary advice (GDA), the LFD demonstrated a substantial decrease in GI symptoms (abdominal pain, bowel movement, and bloating). Following six weeks of LFD compared with GDA, the status of bowel habits, the consistency, and the frequency had statistically substantial improvement. However, the results of these data were more pronounced in patients with IBS-D [74]. Additionally, Patcharatrakul et al. [70] observed an improvement in GI symptoms in 60% of patients who responded after the LFD compared with 28% of patients after brief advice on a commonly recommended diet (BRD) (*p* = 0.001). Following LFD, opposite to BRD, there was a substantial decrease in GI symptoms such as abdominal pain, severity of discomfort, and bloating compared with baseline (*p* > 0.05). 

Ankersen et al. [60] revealed that the LFD decreases the intensity of GI symptoms and also has an effect on bowel habits due to the decrease in stool frequency and an increase in consistency, a factor that was not observed after a diet with a moderate FODMAP diet. Therefore, LFD may be more effective for IBS patients (IBS-D/IBS-M) with frequent soft stools compared with those [76] with less frequent and firm stools (IBS-C).

At the same time, GI symptoms were reduced in patients who received a LFD as in those who followed a moderate-FODMAP diet. Although it was carried out in only 40 patients, a global reduction in symptoms was also noticed in those who received the LFD compared with those who received a diet rich in FODMAP (RR = 0.44; 95% CI: 0.23 up to 0.83) in the study of McIntosh et al. study [67]. 

In various studies conducted by Hustoft et al. [66], LFD was compared with a high-FODMAP diet (FOS), and all GI symptoms improved significantly after 3 weeks of LFD. The most statistically significant improvement included reduced burping (39.4; *p* < 0.001), regurgitation (24.3; *p* < 0.001), and exhaustion (21.2; *p* = 0.001). When those in the LFD group were compared with those in FOS or to those in the placebo group, the placebo group reported a better symptom decrease (80%) compared with that FOS (30%, *p* = 0.13). 

Following LFD dietary intervention compared with the normal diet, Pedersen et al. [71] showed a significantly greater reduction in abdominal pain (OR: 2.97, 95% CI: 1.12–7.89, *p* = 0.03), stool consistency, and frequency (OR: 2.43, 95% CI: 0.97–6.12, *p* = 0.06).

However, patients with IBS were observed for 4 weeks after nutritional intervention in the Guerreiro et al. [65] study that compared LFD to the standard diet (SD). The results demonstrated that the total score for the frequency considerably decreased in both groups compared with the baseline value (LFD: *p* < 0.001; SD: *p* < 0.05), although the LFD group noticed a greater amplitude of the decrease (*p* = 0.041). In terms of treating individual symptoms, it was discovered that an LFD was superior to a SD in relieving abdominal pain and diarrhoea. Although the SD decreased the frequency of constipation, there were no statistically significant differences between these two diets. Furthermore, a questionnaire reported that the LFD group had a 56.4% success rate in improving symptoms overall compared with that of the SD group at 22.2% (*p* = 0.016).

Regarding the effectiveness of the LFD compared with other treatment methods, in the study developed by Menees et al. [68], they evaluated the impact of an LFD versus psyllium treatment. The results revealed that after 4 weeks of LFD intervention, the mean FISI scores for stool consistency improved considerably compared with baseline (39.2 vs. 32.6, *p* = 0.02), but not after psyllium therapy (35.2 vs. 32.5, *p* = 0.22).

In summary, a low-FODMAP diet (LFD) has been shown to have favourable effects on symptoms of irritable bowel syndrome (IBS), particularly in relieving abdominal pain, bloating, and diarrhoea [77]. Improvement in bowel movements and stool characteristics has been reported in several studies, with a significant decrease in the number of bowel movements and an increase in stool consistency. LFDs have been shown to be more effective than a traditional IBS diet, general dietary advice, and a moderate FODMAP diet in reducing GI symptoms. An LFD has also been found to be superior to psyllium therapy in improving stool consistency. In general, an LFD appears to be an effective dietary intervention for managing IBS symptoms.

#### 5.1.2. Effects on Quality of Life in Adults

The LFD substantially improves quality of life for patients with IBS compared with those who follow standard dietary recommendations and a high-FODMAP diet.

The therapeutic effect of an LFD can be measured using the standardised complex score (IBS-SSS). Through it, the frequency and severity of abdominal pain, bloating, frustration with bowel habits, and QoL are measured on a visual analogue scale are measured. Thus, using that score, the positive effect of the LFD was demonstrated by Bohn et al. [65,78], as well as its superiority over a traditional diet. A relevant reduction in total IBS-SSS following the LFD, compared with a low-lactose diet, was also confirmed three years later by Grubel et al. [64,79] in a randomised control trial underlining the importance of patient counselling and supervision by a dietitian. Furthermore, in Hustoft et al. [56], they research reported a mean decrease in IBS-SSS of 163.8 (95% CI: 135.7–500), which was reported [66] after 3 weeks of LFD. Each patient experienced an overall decrease of at least 50 (range: 57–275) [66]. 

The analysis performed by Guerreiro et al. [65] observed an improvement in quality of life after an LFD intervention compared with an SD, as evidenced by an increase in the overall score for quality of life. Compared with baseline, it considerably increased in both groups (LFD: *p* < 0.001; SD: *p* < 0.05), although there was no statistically significant difference between the groups (*p* = 0.2727). However, LFD significantly reduced the negative effects of IBS on dysphoria, interference with daily activities, body image, sexual life, and interpersonal connections with others.

In a study by Naseri et al. [69], the association of LFD with GFD in terms of quality of life was also evaluated. In 73% of the patients, a clinically relevant improvement in IBS-SSS compared with the baseline value was observed after 6 weeks of dietary intervention (*p* = 0.001). In total, 53% of the patients presented a reduction in IBS-SSS of 30 to 60 points after completing the diet, while only 3.3% obtained a decrease of more than 60 points.

Furthermore, three studies by van Lanen et al. [75], Wang et al. [13], and Black et al. [49] evaluated the IBS-SSS score in a meta-analysis carried out on a large sample, more precisely, on 4537 patients from 14 randomised control trials, respectively, 1164 patients from ten randomised control trials, and 944 patients from 13 randomised control trials, observing a mean reduction of 45 points in patients who followed an LFD compared with a control diet; thus, this is consistent with previous studies.

The reviewed studies provide evidence that the low-FODMAP diet (LFD) can substantially improve the quality of life (QoL) of patients with irritable bowel syndrome (IBS) compared with those following standard dietary recommendations or a high-FODMAP diet. The therapeutic effect of LFD can be measured using the standardised IBS-SSS score, which has been used in multiple studies and consistently demonstrated the positive effect of the LFD on reducing abdominal pain, bloating, frustration with bowel habits, and improving QoL. Studies also emphasise the importance of patient counselling and supervision by a dietician during the dietary intervention. Furthermore, LFD was found to significantly reduce the negative effects of IBS on dysphoria, interference with daily activities, body image, sex life, and interpersonal relationships with others. The studies also showed that combining LFD with a gluten-free diet (GFD) can lead to clinically relevant improvements in IBS-SSS score. In general, these studies suggest that LFD can be an effective dietary intervention for patients with IBS to improve their QoL. The review included a total of seven studies.

#### 5.1.3. Effects on Bowel Water Content in Adults

The effects of foods with a higher FODMAP content demonstrated an increase in bowel water content because of the osmotic effect and the increase in gas synthesis by the microbiota in the colon. They exacerbated the symptoms of IBS and functional GI disorders primarily by causing distention and having an osmotic laxative effect.

Studies have shown that fermentable carbohydrates were osmotically active, showing that a diet rich in polyols, sucrose, and fully fermentable carbohydrates caused a doubling of the total wet weight of the effluent due to water retention. With the help of magnetic resonance imaging, it was observed that healthy individuals who drank approximately 18 g of mannitol solution exhibited a 10-fold increase in intestinal water compared with those that drank the same amount of glucose solution. Similar results were also found after 40 g of fructose, with an increase in bowel water compared with the ingestion of 40 g of glucose [80].

Humans have incomplete absorption of fructose and mannitol in the small intestine, leading, through fermentation, to increased gas production in the colon. Increased volume of water in the intestines can worsen abdominal pain and cause diarrhoea [80,81].

The decrease in fructose causes a reduction in the water content in the small intestine, causing a change in the osmotic load in those following an LFD [82].

According to Guerreiro et al. [65], an LFD is beneficial for patients with IBS-D because it decreases osmolarity and, thus, it decreases the water content in the intestinal lumen, which is advantageous in the management of IBS-D. In addition to alleviating symptoms such as abdominal pain and distention that were typically present in all subtypes of IBS, LFD might also help reduce intraluminal fermentation.

On the other hand, Bohn et al. [62] suggested that LFD is the most efficient recommendation to treat IBS, reducing symptoms, healthcare, and social costs [8,26,83,84].

In summary of the five studies considered, we underline and discuss the effects of fermentable carbohydrates on bowel water content and gas synthesis, which exacerbate symptoms of IBS and functional GI disorders. Incomplete absorption of fructose and mannitol in the small intestine leads to increased gas production in the colon, which worsens abdominal pain and causes diarrhoea. Several studies have suggested that an LFD is the most effective recommendation for treating IBS to reduce its symptoms and related healthcare and social costs. LFD benefits patients with IBS-D because it decreases the osmolarity and water content in the intestinal lumen, alleviating symptoms such as abdominal pain and distention. In addition, LFD may help reduce intraluminal fermentation. 

#### 5.1.4. Effects on Biochemical Markers of Disease Activity in Adults 

fCal is an antimicrobial protein secreted primarily by neutrophils, is used for the diagnosis and management of IBS, and is currently preferred due to its specificity over typical inflammatory biomarkers (e.g., CRP). This biochemical marker allows the differentiation of IBS from other organic GI disorders [76].

Following LFD implementation, a decrease in fCal was noticed in the study by Bodini et al. [61] after following a nutritional plan for 6 weeks (T0: 88.4 mg/kg; IQR, 50,220.4 mg/kg vs. T1: 50 mg/kg; IQR, 50.681 mg/kg; *p* = 0.004) compared with that after following a standard diet (T0: 88.4 mg/kg; IQR, 50,220.4 mg/kg vs. T1: 87 mg/kg; IQR, 50,235.6 mg/kg; *p* = 0.175). Therefore, there was a decrease of 34.7% in fCal for patients following LFD compared with 4.4% after a standard diet. This suggests that a low-FODMAP diet may be beneficial in managing IBS symptoms and reducing inflammation in the gut. However, more studies are needed to confirm these findings and determine the long-term effects of a low-FODMAP diet on gut health.

#### 5.1.5. Effects on Nutrient Intake in Adults

Exclusion diets, such as gluten- or dairy-free diets, can cause nutritional deficiencies. The same question was asked about LFDs, regarding the intake of micronutrients.

It was hypothesised that patients following an LFD risked a reduced fibre and micronutrient intake (e.g., calcium, zinc, iron, vitamin D, folic acid, natural antioxidants). 

During a 4-week dietary intervention comparing LFD and mNICE by Eswaran et al. [63], a decrease in the average daily intake of thiamine (*p* = 0.01), riboflavin (*p* = 0.05), calcium (*p* = 0.01), and sodium (*p* = 0.001) was observed; however, that reduction was not sustained after adjusting for energy intake. The causes of the decrease could have been due to the decrease in consumption of grains that typically contained those specific micronutrients. Additionally, calcium intake was low, probably as a result of the limited dairy intake in LFDs. Therefore, substantial micronutrient deficits were not immediately linked to LFDs.

Moreover, Staudacher et al. [78] revealed no significant variations in energy and macronutrients in those who followed the LFD compared to those who followed the control diet. There were indications that the LFD improved overall dietary intake, given the higher vitamin B12 compared with those who followed a standard control diet. 

According to a questionnaire, the LFD was evaluated in the study by Tuck et al. [72] in patients with IBS, whether or not they followed the diet prescribed by a gastroenterologist or dietician. In total, 30% of the patients followed the dietitian’s recommendations, and a specialist did not guide 70%. As a result, it was observed that patients who followed the diet as advised by the dietitian ingested around 12 g of FODMAP (*p* = 0.02), compared with those who were not consulted by a specialist and had lower levels (*p* = 0.04). Furthermore, when each subgroup’s intake was evaluated separately, patients who followed dietician recommendations had significantly fewer polyols than those who did not (*p* = 0.04), which resulted in a decrease in the tendency to consume excess fructose (*p* = 0.08). In terms of micronutrient intake, the group of patients who followed the LFD on the advice of a dietitian had greater values of folate (322 mg vs. 295 mg), iron (13 mg vs. 11 mg), niacin (22 mg vs. 11 mg), and zinc (13 mg vs. 11 mg) compared with those who were not examined by a specialist. The authors concluded that to maintain an optimal intake of micronutrients and macronutrients, patients must be monitored by a dietician through the diet.

This section refers to three studies exploring the potential micronutrient deficiencies associated with the low-FODMAP diet (LFD) for the management of irritable bowel syndrome (IBS). Studies suggest that the LFD may lead to a reduction in the intake of some micronutrients such as thiamine, riboflavin, and calcium, but these reductions were not sustained after adjusting for energy intake. However, the LFD did not cause significant micronutrient deficits, and there were indications that it improved overall dietary intake. Patients who followed the LFD under the guidance of a dietitian had a greater intake of folate, iron, niacin, and zinc compared with those who did not receive specialist advice. Therefore, to maintain an optimal intake of micronutrients and macronutrients, patients should be monitored by a dietitian while following the LFD.

### 5.2. Results and Discussion in Children

Using the databases the Cochrane Central Register of Controlled Trials (CENTRAL), the Cochrane Database of Systematic Reviews (CDSR), the Excerpta Medica Database (EMBASE), Medline, PubMed, Scopus, and Web of Science, four randomised control trials were identified. The main characteristics of the investigations are summarised in Table 3.

#### 5.2.1. Effects on Global Symptoms, Abdominal Pain, and Bloating in Children

In recent years, the effectiveness of LFDs has also been evaluated among children (age 4–18 years). Unfortunately, we have identified few randomised control trials that have shown the effectiveness of that diet, in a small number of patients.

Functional abdominal pain is a common paediatric GI disorder characterised by chronic or recurrent abdominal pain that is not associated with any structural, inflammatory, or metabolic causes [88,89].

One of the studies on children, published by Boradyn et al. [79] in 2020, divided the subjects into two categories: the first group was represented by those who followed an LFD, and the second group followed the diet recommended by NICE. The results of the randomised control trial did not show a significant reduction in symptoms after LFD compared to the diet recommended by NICE. The study was carried out on 171 parents of children diagnosed with functional abdominal pain to assess their opinion about the low-FODMAP diet. The results showed that while 70% of parents had never heard of the diet before, after being informed about it, most were willing to try it as a dietary intervention for their children’s functional abdominal pain (FAP). However, parents also expressed concerns about the complexity and feasibility of the diet, as well as the potential risk of nutrient deficiencies. In general, the study suggested that parental opinion and support play an important role in the success of the low-FODMAP diet as a dietary intervention for children with FAP. The findings also highlighted the need for healthcare professionals to provide clear information and support to parents considering the low-FODMAP diet as a dietary intervention for their children’s FAP [88].

Nogay et al. [87] evaluated, for the first time, the effectiveness of LFD on GI and behavioural issues in children with autism spectrum disorders (ASD) (e.g., self-harm, repetitive behaviour, screaming, anxiety), considering the strong impact that behavioural problems play in the etiopathogenesis of GI problems. Rhys-Jones et al. [20] evaluated through meta-analysis the use of the LFD in paediatrics and its impact on macronutrient intake by analysing five randomised control trials. The research results identified a valid decrease in the frequency and consistency of stool in children diagnosed with IBS. Those improvements due to the decrease in carbohydrate intake were visible in the first few weeks of the diet. However, implementing the LFD among children raised some concerns about the intake of nutrients since the LFD is a restrictive food diet. There were no discernible differences between the groups that followed the LFD and the control diet in terms of nutrient intake, except for vitamins B12 and K. Vitamin B12 had lower levels for those who followed the LFD compared with the group that followed the control diet, most likely as a result of the decrease in dairy product intake. Some reported a lower calcium intake due to reduced consumption of certain dairy products or a decrease in vitamin B2 and increased levels of vitamin B3 and vitamin B6 in the group that followed the LFD compared with a control diet for 4 weeks. In some cases, the intake of vitamin C, vitamin B6, and vitamin E was improved by supplementing the portions of vegetables and proteins in the paediatric dietitian.

In addition to all those highlighted aspects, regarding the intake of macronutrients for children, additional studies are necessary to assert whether the temporary restriction of FODMAPs impacts the child’s harmonious growth and development. According to the text, there is limited evidence from RTCs to support the effectiveness of the LFD in the treatment of functional abdominal pain in children. Additional studies are necessary to determine the impact of temporary FODMAP restrictions on children’s balanced growth and development.

#### 5.2.2. Effects on Quality of Life in Children

Most studies performed on adults use IBS-SSS to evaluate quality of life, but it does not apply to paediatrics.

El Gendy et al. [85] evaluated the quality of life based on the KIDSCREEN-10 index to assert the subjective health and well-being of children and adolescents. It was created as a self-reporting tool, which is easily applicable to healthy and chronically ill children and adolescents. The LFD food intervention in the research of El Gendy et al. [85] showed a decrease in the pain score for 84% of the patients, where the median score at the beginning of the study was 8 (IQR: 6–10) in the range of 4–10. After two months of the diet, it had a value of 4 (IQR: 4–6) and the range was between 0–10 (*p* = 0.0000). That pain reduction in children was later associated with an improved quality of life. Therefore, the LFD demonstrated a reduction in intestinal pain and quality of life in young patients with functional abdominal pain and even showed a positive increase in weight among children and adolescents because the diet was carefully monitored by a paediatric dietitian specialised in gastroenterology to ensure optimal intake of calories, vitamins, and minerals appropriate for their age and constitution [85]. 

Boradyn et al. [79] showed that parents perceived functional abdominal pain to have a significant impact on their children’s quality of life, and many reported that their children had missed school or social activities due to their symptoms, so by alleviating those symptoms they perceived the diet to have a positive impact on their children’s quality of life. 

Nogay et al. [87] revealed that preschoolers with ASD had a higher prevalence of GI symptoms, such as abdominal pain and bloating, compared with typically developing children. That study also found a significant relationship between GI symptoms and behavioural problems in preschoolers with ASD, such as irritability and hyperactivity. Furthermore, the severity of GI symptoms was found to be related to the severity of ASD symptoms, suggesting that there may be a complex interplay between GI symptoms and ASD symptoms. Overall, the study highlighted the importance of addressing GI symptoms in preschoolers with ASD, as they could significantly impact the child’s behaviour and quality of life. It also suggested that there might be a need for more comprehensive medical evaluations and interventions to address GI symptoms in children with ASD. In this text, three studies are considered. In summary, these studies highlight the importance of addressing GI symptoms in children and adolescents, as they could significantly impact their behaviour and quality of life. It is worth mentioning that only three studies were taken into account in this text.

#### 5.2.3. Effects on Bowel Water Content in Children

Children with IBS may experience alterations in water content, which can contribute to their symptoms [90,91]. Research studies have found that children with IBS had a lower stool water content compared with healthy children, indicating that their faecal material was drier and harder to pass. Additionally, low water intake had been associated with a higher severity in children with IBS. The altered bowel water content in children with IBS could have been related to underlying factors such as abnormal intestinal permeability, changes in the intestinal microbiota, and increased levels of nitric oxide in the intestine. Understanding these factors might help develop targeted treatment strategies to alleviate symptoms in children with IBS [21,92]. A study used endoscopy to obtain rectal biopsies of children with IBD and healthy children. The biopsies were then analysed for the expression of nitric oxide synthases enzymes and the presence of nitric oxide. They found that children with IBD had increased expressions of nitric oxide synthase enzymes and higher levels of nitric oxide in their rectal mucosa compared with healthy children. Higher levels of nitric oxide were associated with an increase in water content in children with IBD. The authors concluded that nitric oxide played an important role in the pathogenesis of IBD in children and may contribute to intestinal dysfunction by altering water content [93].

Therefore, understanding the underlying factors that contribute to altered intestinal water content can help develop targeted treatment strategies to alleviate symptoms in children with IBS and IBD.

#### 5.2.4. Effects on Biochemical Markers of Disease Activity in Children

The potential use of these markers in diagnosing, assessing disease activity, and predicting outcomes in patients with IBD is of high interest. 

A study conducted by Fodor et al. [94] discussed the limitations and challenges associated with these markers, including the lack of specificity and sensitivity of some markers and the need for standardised assays and interpretations. Furthermore, emerging markers, including faecal biomarkers, genetic markers, and microbiome-related markers, have been discussed that have shown promise in recent studies for the diagnosis and monitoring of IBD. Overall, the article concluded that the use of biochemical markers in the management of IBD is still evolving, and further research is needed to identify specific and reliable and specific markers that can be used in clinical practise [94].

The research carried out by Joishy et al. [86] discusses the use of faecal calprotectin and lactoferrin as non-invasive markers of inflammatory bowel disease (IBD), including Crohn’s disease and ulcerative colitis, in children. The study found that faecal calprotectin and lactoferrin were reliable markers for the detection of IBD, with high sensitivity and specificity, and that they could differentiate between IBD and other non-inflammatory bowel conditions such as IBS. 

In general, more research is needed to identify reliable and specific markers that can be used in clinical practise. Two studies were taken into account.

#### 5.2.5. Effects on Nutrient Intake in Children

The study of Boradyn et al. [79] revealed that, while parents were generally willing to try the diet as a dietary intervention for their children’s functional abdominal pain, they also expressed concerns about the possible risk of nutrient deficiencies. The article suggests that while LFD may effectively reduce symptoms of functional abdominal pain in children, it is important to ensure that the diet is nutritionally adequate and does not cause nutrient deficiencies. It is recommended that the LFD be implemented under the guidance of a healthcare professional, which can ensure that the diet is nutritionally adequate and that the child’s nutritional status is monitored. Overall, the research highlights the importance of ensuring that any dietary intervention implemented in children with FAP effectively reduces symptoms and does not lead to nutritional deficiencies [79]. 

Nogay et al. [91] suggested that nutritional deficiencies can play a role in the development and severity of symptoms of ASD and that addressing nutritional deficiencies can positively impact the behaviour and development of children with ASD. However, the article does not provide a detailed analysis of nutritional intake or specific nutrient deficiencies in preschoolers with ASD. It is highlighted that adequate nutrition is needed while implementing nutritional interventions for children with functional abdominal pain and autism spectrum disorder. Overall, in the two studies considered, it is crucial to ensure that any dietary interventions implemented in children with health conditions effectively reduce symptoms and do not lead to nutritional deficiencies.

## 6. Conclusions

In conclusion, a diet that restricts the intake of fermentable oligosaccharides, disaccharides, monosaccharides, and polyols could be a feasible approach to the management of irritable bowel syndrome to improve abdominal symptoms (e.g., discomfort, pain, flatulence, bloating) and the quality of life in adults and children. 

However, the low-FODMAP diet raises some challenges regarding the alteration of the intestinal microbial flora and the lack of nutrients in the absence of guidance from a dietitian. Therefore, randomised controlled trials with larger sample sizes and longer follow-ups are needed to confirm the superiority of this diet over others; additionally, more studies on the effects in children are also necessary.

The purpose of our review was to provide comprehensive data on the results of following a low-FODMAP diet, summarised in separate sections for the main symptoms of IBS, in order to better promote this diet as a viable management option for the various types of IBS. 

## Figures and Tables

**Table 1:** Products with high and low-FODMAP content.

Food Products	High-FODMAP Content	Low-FODMAP Content
vegetables	asparagus, garlic, onions, broccoli, green peas, sugar snap peas, mushrooms, cabbage	capsicum, carrot, corn, cucumber, eggplant, green beans, lettuce, pumpkin, tomato, zucchini
fruits	apples, pears, mangos, watermelon, nectarines, peaches, plums, dried fruits	orange, mandarin, grapes, blueberries, lemon, kiwi, banana, strawberries
dairy and alternatives	milk (cow, goat, sheep), condensed milk, yoghurt, cream, ice cream, cheese (fresh), soy milk	lactose-free milk, almond/rice milk, lactose-free yogurts, ripened cheese, peanut butter, hard cheese, camembert/brie cheese
bread and cereals	rye, wheat-containing bread, wheat-based cereals with dried fruit, wheat pasta, breakfast cereals	rice, quinoa, gluten-free bread, gluten-free pasta, sourdough, spelt bread
nuts and seeds	pistachios and cashews	peanuts, walnuts, pumpkin seeds

**Table 2:** Characteristics of randomised control trial (RCT) studies in adults.

Author	Type	Size of the Study	Study Characteristics	Conclusions
Ankersen et al. [60]	RCT	*n* = 29	Adults diagnosed with IBS according to Rome IV criteria. Comparing LFD with a moderate FODMAP diet. Exclusion criteria: patients with previous GI surgery, cardiovascular, liver, psychiatric, and neurological diseases, and other GI disease; patients with allergies or intolerance to food; and patients who used antibiotics within a month before the start of the trial.	LFD decreased the intensity of GI symptoms, including less frequent and firmer stool, when compared with moderate portions of the FODMAP diet. LFD seemed more helpful for IBS patients (IBS-D/IBS-M) with frequent loose stools than those with IBS-C.
Bodini et al. [61]	RCT	*n* = 127	RCT with adults diagnosed with IBS, according to Rome IV criteria, compares LFD with SD. Exclusion criteria: patients with moderate to severe disease, patients with previous GI surgery, and patients with coeliac disease, diabetes, and lactose intolerance.	The study highlighted the impact of LFD on the treatment of IBS and other intestinal diseases by evaluating some intestinal inflammatory markers (fCal and CRP dose in the beginning and after 6 weeks of the nutrition plan). A decrease in faecal biomarkers was observed, which was also associated with improvements in QoL.
Bohn et al. [62]	RCT	*n* = 67	RCT with adults diagnosed with IBS according to Rome III criteria compared LFD with NICE. Exclusion criteria: patients with cardiac, neurological, liver, psychiatric, or IBD.	The study showed that offering food guidance to patients with IBS in a medical environment helped improve GI symptoms; however, there were no obvious distinctions between LFD and NICE, as both reduced IBS symptoms.
Eswaran et al. [63]	RCT	*n* = 84	RCT with adults diagnosed with IBS-D according to Rome III criteria, compared LFD with mNICE. Exclusion criteria: patients with IBS-C, GI diseases, IBD, patients with previous GI surgery, pregnant patients, and patients using antibiotics or narcotics within a month before the beginning of the trial.	During a 4-week nutritional intervention, the low-FODMAP diet significantly exceeded the mNICE diet to improve disease-specific QoL across all dimensions of the IBS-QoL questionnaire, except eliminating food. Following the introduction of LFD, a decrease in the average daily consumption of some micronutrients was observed, although there were no changes in the amount of energy consumed. Therefore, LFD was not immediately associated with significant nutritional deficits.
Grubel et al. [64]	RCT	*n* = 39	RCT with adults diagnosed with IBS, according to Rome IV criteria, which compared LFD with a low-lactose diet. Exclusion criteria: patients with coeliac disease, patients with food allergies, and patients using laxatives, antidiarrheal agents, and antibiotics.	LFD was associated with significantly fewer IBS symptoms than a low-lactose diet, highlighting the susceptibility of short-chain carbohydrates to poor digestion. That improvement was also due to the advice of the dietitian. Pain severity/frequency, bloating, and stool habits had better subscores when following an LFD.
Guerroiro et al. [65]	RCT	*n* = 70	A clinical trial with adult patients with IBS according to Rome IV criteria. Comparing LFD with SD. Exclusion criteria: patients with previous GI diseases and surgery, patients using antibiotics, prebiotics, and probiotics within a month before the start of the trial.	The global symptom frequency scores of both groups decreased significantly compared with baseline. However, the LFD group had a greater decrease in magnitude. LFD has been suggested to be more efficient than SD in reducing pain and diarrhoea. Although SD decreased the frequency of constipation, there were no statistically significant differences between the diets. Furthermore, the overall score for QoL increased significantly in both groups compared with baseline, with no statistically significant differences between the groups.
Hustoft et al. [66]	RCT	*n* = 20	A clinical trial with adult patients with IBS-D or IBD-M according to Rome III criteria, comparing LFD with FOS. Exclusion criteria: patients with IBS-C, pregnant women, and patients using probiotics or antibiotics.	In patients diagnosed with IBS-D or IBS-M, LFD was best at decreasing functional GI symptoms, and significantly more participants had symptom relief in response to a placebo (80%) than FOS (30%).
McIntosh et al. [67]	RCT	*n* = 37	According to Rome III criteria, a clinical trial of adult patients with IBS compares LFD with a high-FODMAP diet. Exclusion criteria: patients with previous GI surgery, patients using antibiotics, stool bulking agents, narcotics, or lactulose.	After 3 weeks, comparing patients diagnosed with IBS who received LFD with those who received a high-FODMAP diet, an overall decrease in GI symptoms was observed.
Menees et al. [68]	RCT	*n* = 43	According to Rome III criteria, adults diagnosed with IBS compare the effectiveness of an LFD vs. psyllium. Exclusion criteria: patients with dementia, diabetes, scleroderma, IBD, renal and hepatic disease, patients with previous GI surgery, and patients using antibiotics, prebiotics, probiotics, or narcotics.	The proportion of patients who reported a decrease of 50% in global symptoms was comparable for both groups. The psyllium group revealed a greater improvement in overall symptoms, but the LFD group reported a better QoL and stool consistency.
Naseri et al. [69]	RCT	*n* = 42	According to Rome IV criteria, adults diagnosed with IBS associated LFD with GFD. Exclusion criteria: patients with coeliac disease, IBD, liver disease, patients with precedent GI surgery, cancer, and patients using NSAIDs and drinking alcohol.	IBS patients who ingested LFD with GFD saw a substantial decrease in IBS symptoms and an adjustment of their gut microbiome. Intestinal inflammation can be reduced by association, which decreases IBS-SSS.
Patcharatrakul et al. [70]	RCT	*n* = 62	Adults diagnosed with IBS according to Rome III criteria, with moderate to severe GI symptoms, comparing LFD with BRD. Exclusion criteria: patients with previous GI surgery; coeliac disease; GI cancers; severe cardiovascular, liver, lung, neurological or mental diseases; and patients who used antibiotics, prebiotics, probiotics, or symbiotics within a month before the start of the study.	Compared with the BRD diet, the LFD proved its efficiency in decreasing VAS values. Following the LFD intervention, abdominal discomfort and bloating decreased considerably from their baseline values compared with those who received BRD. After both approaches, there were no significant improvements in belching or stool urgency.
Pederson et al. [71]	RCT	*n* = 123	A clinical trial of adult patients with IBS according to Rome III criteria, comparing LFD with ND. Exclusion criteria: pregnant women, patients with GI surgery.	After 6 weeks of dietary intervention, patients who followed LFD compared with ND had a significant reduction in the IBS-SSS average.
Tuck et al. [72]	RCT	*n* = 80	A questionnaire was used to gather information about how LFD impacts patients with IBS.	Half of the patients reported an improvement in GI symptoms, but many did not reach the therapeutic level of FODMAP intake level, especially in the absence of the diet physician’s guidance.
Wong et al. [73]	RCT	*n* = 16	Adults diagnosed with IBS according to Rome III criteria analyse the impact of LFD in Asian patients. Exclusion criteria: patients with frequent organic diseases (cancer and inflammatory bowel disease).	11 of 16 patients (68.8%) reported an improvement in their general symptoms, which were classified in the following order: abdominal pain (60%), bloating / distension (70%), and flatulence (87.5%).
Zahedi et al. [74]	RCT	*n* = 101	According to Rome III criteria, the study involved the clinical response in patients with IBS-D after LFD vs. GDA. Exclusion criteria: patients with coeliac disease; IBD; cardiovascular, liver, kidney, and neurological diseases; diabetes; and thyroid disorders.	After six weeks, patients with IBS-D had a satisfactory reduction in GI symptoms with both LFD and GDA. However, LFD had greater benefits in improving IBS, such as a reduction in the severity, frequency, and status of abdominal pain and abdominal distention. However, in contrast with the GDA group, LFD did not affect quality of life.

Abbreviations: Brief advice on a commonly recommended diet (BRD), high fructose-oligosaccharide diet (FOS), GI (GI), general dietary advice (GDA), gluten-free diet (GFD), inflammatory bowel disease (IBD), irritable bowel syndrome (IBS), irritable bowel syndrome with diarrhoea (IBS-D), modified National Institute of Health and Clinical Excellence dietary intervention (mNICE), normal diet (ND), non-steroidal anti-inflammatory drugs (NSAIDs), randomised control trial (RCT), standardised complex score (IBS-SSS), standard diet (SD), visual analogue scale (VAS), quality of life (QoL).

**Table 3:** Characteristics of randomised control trial (RCT) studies in children.

Author	Type	Size of Study	Study Characteristics	Conclusions
Boradyn et al. [79]	RCT	*n* = 29	RCT with a parenteral opinion about LFD on children (age: 5–12 years) diagnosed with FAP, according to Rome III criteria. Exclusion criteria: patients with organic GI disorders, patients with food allergies, patients with acute infection, and patients with antibiotics, within two months of starting the study.	The effectiveness of LFD was evaluated after 4 weeks of dietary intervention based on parents’ opinions on the intensity of their children’s abdominal pain. LFD and BDA/NICE diets required the supervision of a paediatric dietician to obtain an effective result in children, thus avoiding nutritional deficiencies.
El Gendy et al. [85]	RCT	*n* = 50	RCT evaluated the effects of LFD in children (age: 3–18 years) diagnosed with FAP, according to Rome IV. Exclusion criteria: patients with a family history of IBD, coeliac disease, peptic ulcer disease, dysphagia, vomiting, blood loss, odynophagia, diarrhea, arthritis, and weight loss.	After 2 months of LFD intervention, a decrease in pain intensity was observed in 74% of the patients, as well as an increase in quality of life, without detrimental effects on body weight.
Joishy et al. [86]	RCT	*n* = 74	The RCT evaluates faecal calprotectin and lactoferrin in children (age 4–17 years) with IBD.	Faecal calprotectin and lactoferrin were evaluated as highly precise and non-invasive indicators for the preliminary identification of inflammatory bowel disease (IBD), Crohn’s disease, and ulcerative colitis in children. They could help distinguish between IBD and other non-inflammatory bowel diseases such as IBS.
Nogay et al. [87]	RCT	*n* = 15	RCT evaluating the effect of LFD in children (age: 6–17 years) with ASD together with IBS according to Rome IV. Exclusion criteria: patients with previous GI surgery, patients with IBD, cystic fibrosis, liver and cardiovascular disease, and patients using antibiotics.	After 2 weeks, the LFD intervention had benefits in children diagnosed with autism with abdominal pain and/or constipation, as it was effective in reducing constipation and other GI problems without affecting the intake of nutrients.

Abbreviations: autism spectrum disorder (ASD), functional abdominal pain (FAP), gastrointestinal (GI), inflammatory bowel disease (IBD), irritable bowel syndrome (IBS), randomised control trial (RCT), standard diet (SD), quality of life (QoL).
